# Kaemphenolide: a cyclobutane-bearing phenylpropanoid from *Kaempferia galanga* L. with nitric oxide inhibitory activity

**DOI:** 10.1007/s13659-025-00547-2

**Published:** 2025-10-02

**Authors:** Syarifatul Mufidah, Yusaku Miyamae, Hiroyuki Fuchino, Nobuo Kawahara

**Affiliations:** 1 Master’s/Doctoral Program in Life Science Innovation, University of Tsukuba, Tsukuba, Japan; 2 Faculty of Pharmacy, Universitas Ahmad Dahlan, Yogyakarta, Indonesia; 3 Institute of Life and Environmental Sciences, University of Tsukuba, Tsukuba, Japan; 4 Research Center for Medicinal Plant Resources, National Institutes of Biomedical Innovation, Health and Nutrition, Tsukuba, Japan; 5 Present Address: Faculty of Pharmacy, Niigata University of Pharmacy and Medical and Life Sciences, Niigata, Japan; 6 Present Address: The Kochi Prefectural Makino Botanical Garden, Kochi, Japan

**Keywords:** Phenylpropanoid, Cyclobutane, *Kaempferia galanga*, Anti-inflammation

## Abstract

An undescribed phenylpropanoid dimer featuring a rare cyclobutane ring was isolated from the rhizomes of *Kaempferia galanga* L., a medicinal plant traditionally used in Southeast Asia for its anti-inflammatory and therapeutic properties. Kaemphenolide (**1**), along with five known constituents, was obtained by separations of methanolic extract using chromatography. The presence of a cyclobutane ring within the phenylpropanoid scaffold represents an unusual structural motif among natural products and underscores the chemical uniqueness of this molecule. To evaluate its biological relevance, **1** was tested for its ability to inhibit nitric oxide (NO) production in lipopolysaccharide (LPS)-stimulated RAW264.7 macrophages. The compound exhibited anti-inflammatory activity, with an IC_50_ value of 23.1 ± 6.40 µM.

**Graphical Abstract:**

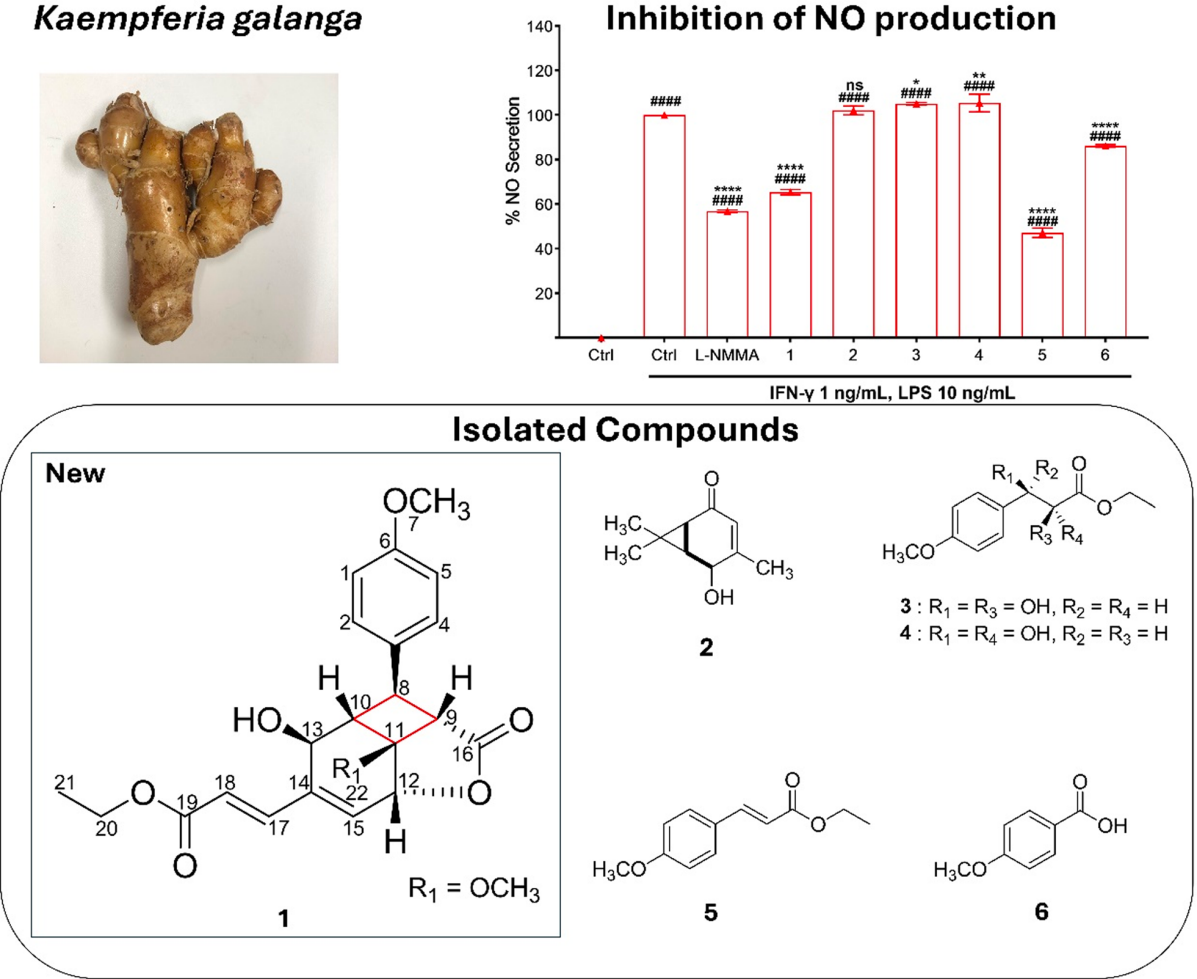

**Supplementary Information:**

The online version contains supplementary material available at 10.1007/s13659-025-00547-2.

## Introduction

*Kaempferia galanga* L. (*Zingiberaceae*) is a prominent medicinal and culinary plant widely utilized throughout Southeast Asia, notably in Indonesia, China, and Malaysia [1, 2]. Traditionally, it has been employed to address a range of ailments [3], including inflammation [4], hypertension [5], headaches [6], and digestive disorders [7]. The rhizome of KG is also a key ingredient in traditional remedies such as jamu beras kencur in Indonesia [8]. A review by Wang et al. documented the isolation of 97 compounds from *K. galanga* between 1987 and 2020, underscoring the plant’s significant chemical diversity and its sustained potential as a source of bioactive natural products [9].

Phenylpropanoids represent a structurally and pharmacologically significant class of compounds identified within diverse natural product [10]. A recent study by Zhu et al. (2024) has highlighted the presence of six new phenylpropanoids isolated from *K. galanga*, including four containing cyclobutane rings formed through [2 + 2] cycloaddition [11]. Interestingly, only one of those compounds exhibited notable anti-inflammatory activity, indicating that structural diversity may contribute to the varied biological profile. Cyclobutane-containing natural products possess a unique structural characteristic due to the inherent ring strain and biosynthetic challenges of forming four-membered carbocycles [12]. Despite their rarity, these frameworks often confer distinct biological activities, positioning them as promising scaffolds in drug discovery [13]. The cyclobutane motif, though relatively rare among natural products, appearing in fewer than 0.1% of known structures due to its high ring strain and biosynthetic complexity [12] remains a valuable scaffold that inspires synthetic efforts, particularly through [2 + 2] cycloaddition strategies [14, 15], to construct or optimize structurally intricate and biologically active compounds. Here, we describe the isolation of six isolated compounds including a cyclobutane-containing phenylpropaoid from *K. galanga*, along with an evaluation of its anti-inflammatory activity.

## Results and discussion

A phenylpropanoid bearing cyclobutane ring, compound **1**, was isolated as a yellow oil from the ethyl acetate fraction. Thin-layer chromatography (TLC) analysis revealed an *R*_*f*_ value of 0.3 in a solvent system of *n*-hexane:ethyl acetate (1:1), with absorption observed under UV Light at 254 nm. Upon heating post-derivatization with 10% H_2_SO_4_, the compound exhibited an orange coloration under 366 nm UV light. High-resolution ESI-Orbitrap-MS in positive ion mode displayed a molecular ion peak at *m/z* 401.15958 [M + H]^+^, consistent with a molecular formula of C_22_H_24_O_7_.

Infrared (IR) spectroscopy indicated the presence of a hydroxyl group through a broad absorption band at 3421 cm⁻^1^ and a carbonyl group at 1714 cm⁻^1^. The ^1^H NMR spectrum (Table 1) of compound **1** showed signals attributable to four aromatic protons [*δ*_H_ 7.19 (2H, *J* = 8.5 Hz), 6.90 (2H, *J* = 8.5 Hz)], consistent with a 1,4-disubstituted benzene ring; a pair of trans-olefinic protons [*δ*_H_ 7.38, 6.26, each *J* = 16 Hz]; a single olefinic proton (*δ*_H_ 6.41); two oxymethine protons (*δ*_H_ 5.15, 4.46); two methoxy groups (*δ*_H_ 3.81, 3.40); an oxygenated ethyl moiety (*δ*_H_ 4.26, q, 2H; 1.32, t, 3H); and several other resonances corresponding to methine protons.

**Table 1:** ^1^H- and ^13^C-NMR spectral data of compounds **1** and **1a**

No	**1** (CDCl_3_, 600 MHz)		**1a**^27^ (300 MHz)
	*δ*_H_	*δ*_C_	*δ*_H_
1	6.90 (d, *J* = 8.5 Hz, 2H)	114.4 CH	6.89
5			
2	7.19 (d, *J* = 8.5 Hz, 2H)	127.7	7.18
4			
3	–	131.7	–
6	–	159.1	–
7	3.81 (s, 3H)	55.4	3.81 (s, 3H)
8	2.67 (dd, *J* = 8.9, 7.3 Hz, 1H)	36.5	2.66 (dd, *J* = 9, 7 Hz, 1H)
9	3.22 (d, *J* = 7.1 Hz, 1H)	48.1	3.21 (d, *J* = 7 Hz, 1H)
10	3.29 (dd, *J* = 9.0, 2.0 Hz, 1H)	47.6	3.28 (dd, *J* = 9, 2 Hz, 1H)
11	–	75.2	–
12	5.15 (d, *J* = 3.8 Hz, 1H)	77.9	5.14 (d, *J* = 4 Hz, 1H)
13	4.46 (d, *J* = 1.9 Hz, 1H)	63.2	4.46 (brdd, *J* = 5, 2 Hz, 1H)
14	–	136.7	–
15	6.41 (d, *J* = 3.8 Hz, 1H)	131.7	6.41 (d, *J* = 4 Hz, 1H)
16	–	175.1	–
17	6.26 (d, *J* = 16.0 Hz, 1H)	121.5	6.23 (d, *J* = 16 Hz, 1H)
18	7.38 (d, *J* = 16.0 Hz, 1H)	143.1	7.35 (d, *J* = 16 Hz, 1H)
19	–	166.2	–
20	4.26 (q, *J* = 7.1 Hz, 2H)	60.9	4.21 (t, *J* = 7 Hz, 2H)
21	1.32 (t, *J* = 7.1 Hz, 3H)	14.3	1.56 (dt, *J* = 7, 7 Hz, 2H)
22	3.40 (s, 3H)	51.9	3.40 (s, 3H)
23	–	–	1.71 (tqq, *J* = 7, 7, 7 Hz, 1H)
24/25	–	–	0.93 (d, *J* = 7 Hz, 6H)

The ^13^C NMR spectrum (Table 1) exhibited 20 carbon signals (with two overlapping), including two ester carbonyls (*δ*_C_ 175.1, 166.2), eight *sp*^2^ carbons (including overlapping aromatic carbons), and five oxygenated carbons [*δ*_C_ 77.9, 63.2, 60.9; and methoxy carbons at *δ*_C_ 55.4, 51.9]. The HMQC experiment confirmed direct proton-carbon correlations, as listed in Table 1.

**Fig. 1:**
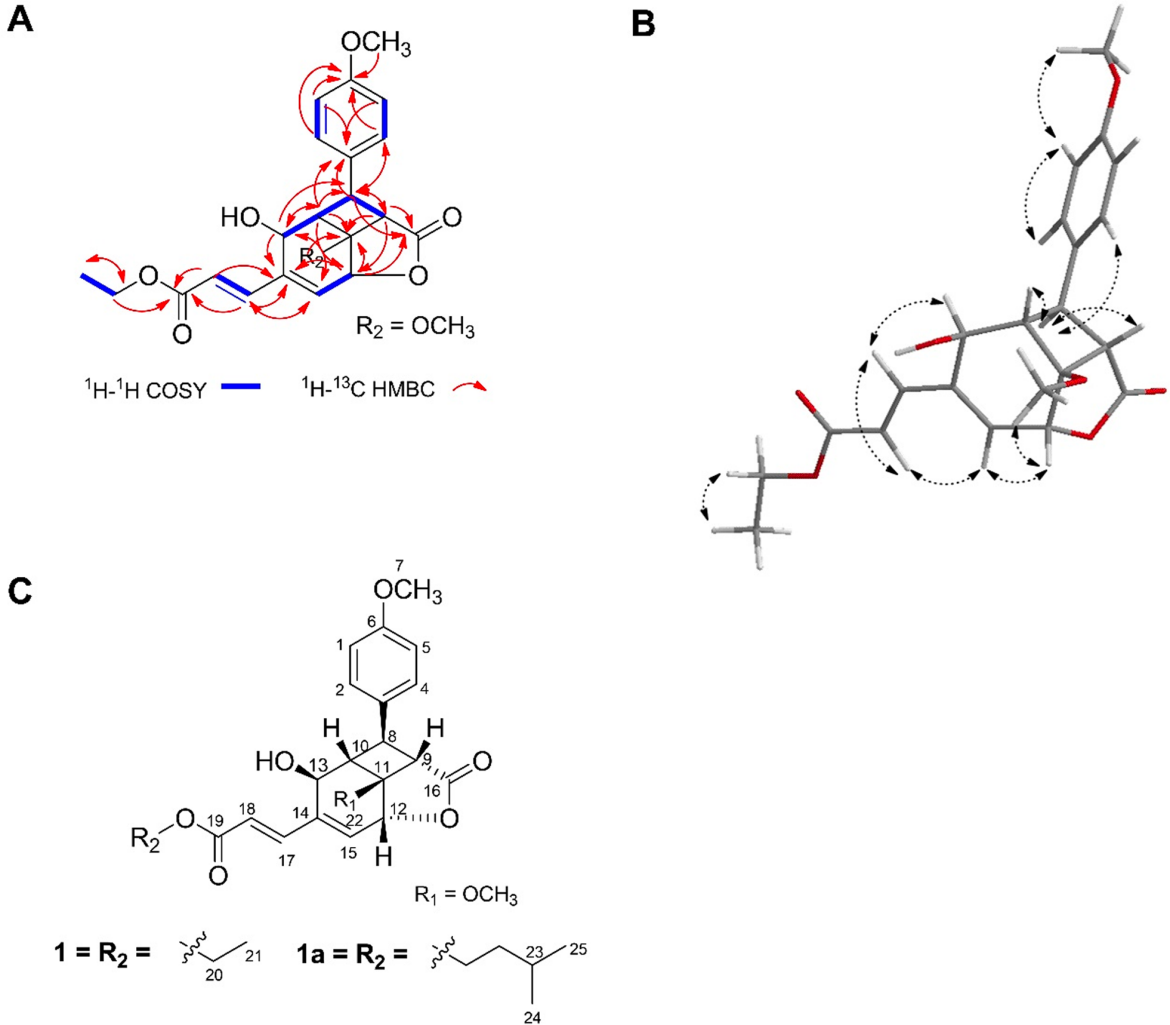
Compound **1**. **A** ^1^H–^1^H COSY and HMBC correlations; **B** ^1^H–^1^H NOESY correlation; **C** Comparison of compounds **1** and **1a**

The COSY spectrum demonstrated proton coupling within the aromatic system [H-1/H-5 (*δ*_H_ 6.90), H-2/H-4 (*δ*_H_ 7.19)], between oxymethine protons H-12 (*δ*_H_ 5.15) and H-15 (*δ*_H_ 6.41), between the ethyl protons H-20 (*δ*_H_ 4.26) and H-21 (*δ*_H_ 1.32), as well as between the trans-olefinic protons H-17 (*δ*_H_ 7.38) and H-18 (*δ*_H_ 6.26). Evidence for the presence of a cyclobutane ring was indicated by the deshielded resonances and correlations involving H-8 (*δ*_H_ 2.67), H-10 (*δ*_H_ 3.29), and H-9 (*δ*_H_ 3.22), with further support from HMBC correlations between H-9/C-11 and H-10/C-11 (Fig. 1A). Further HMBC correlations supported the structural connectivity between the cyclobutane ring, a lactone, and a cyclohexane moiety. Specifically, correlations were observed between H-8/C-16, H-9/C-12, H-12/C-16, H-12/C-11, H-12/C-14, H-13/C-8, and H-10/C-12. The first methoxy group [*δ*_H_ 3.81, *δ*_C_ 55.4] was attached to the aromatic ring, evidenced by HMBC correlations between H-7 and C-6. The second methoxy group [*δ*_H_ 3.40, *δ*_C_ 51.9] was linked to the cyclobutane ring, as shown by the HMBC correlation between H-22 and C-11.

The planar structure of compound **1** was elucidated by extensive 2D NMR analysis (Fig. 1A). HMBC correlations between H-8/C-3, H-8/C-4, and H-2/4/C-8 further confirmed the linkage of the cyclobutane to the methoxyphenyl moiety. The ethyl acrylate unit was found to be appended to the cyclohexane ring at its olefinic side, supported by HMBC correlations involving H-18/C-14, H-17/C-19, H-18/C-19, H-17/C-15, and H-20/C-19.

A structurally similar compound, **1a**, was previously reported by A. Schrader et al. as a photochemical product [16]. The primary structural divergence between **1** and **1a** lies in the nature of their alkyl ester substituents (Fig. 1B). NOESY spectra provided additional stereochemical insight, revealing cross-peaks between H-7 and H-1/5, H-12 and H-22, H-13 and H-18, and H-15 and H-17. However, due to overlapping resonances within the cyclobutane region, a 1D-difference NOE experiment was performed to supplement the configuration assignment.

Upon selective irradiation of H-8 (*δ*_H_ 2.67), enhancements were observed for H-9 (*δ*_H_ 3.22), H-13 (*δ*_H_ 4.46), and H-2/4 (*δ*_H_ 7.19). Comparative 1D-difference NOE data between **1** and **1a** are summarized in Table 2. Notably, compound **1** showed reciprocal NOE enhancements between H-8 and H-9, although at low intensities, which may be attributed to differences in NMR instrumentation-compound **1** being measured on a 600 MHz spectrometer, while **1a** was analyzed on a 300 MHz system. Consequently, minor signal enhancements may have arisen from interactions with neighboring protons. Overall, the relative stereochemistry of compound **1** mirrors that of compound **1a**, as depicted in the complete structural representation (Fig. 1B) and further supported by the three-dimensional conformational model (Fig. 1C).

**Table 2:** 1D difference NOE data of compounds **1** and **1a**

Selective proton	**1**	**1a**
	Irradiate protons	
H-13	H-8 (7%), H-10 (3%), H-18 (16%)	H-8 (6%), H-10 (7%), H-18 (20%)
H-10	H-2/4 (6%), H-13 (8%)	H-2/4 (5%), H-13 (5%)
H-12	H-15 (6%)	H-15 (8%), H-22 (5%)
H-8	H-2/4 (5%), H-13 (3%), H-9 (3%)	H-2/4 (5%), H-13 (4%)
H-9	H-2/4 (5%), H-8 (4%)	H-2/4 (5%)
H-22	H-12 (3%), H-10 (2%), H-9 (2%)	H-12 (10%), H-9 (5%)

Several known compounds were also isolated and identified through spectroscopic comparisons with Literature data, including 3-caren-5-one-2-ol (**2**) [17], ethyl-*syn*-2,3-dihydroxy-3-(4-methoxyphenyl) propanoate (**3**) [18], ethyl-*anti*-2,3-dihydroxy-3-(4-methoxyphenyl) propanoate (**4**) [18], ethyl-4-methoxycinnamate (**5**) [19], 4-methoxybenzoic acid (**6**) [20].

**Fig. 2:**
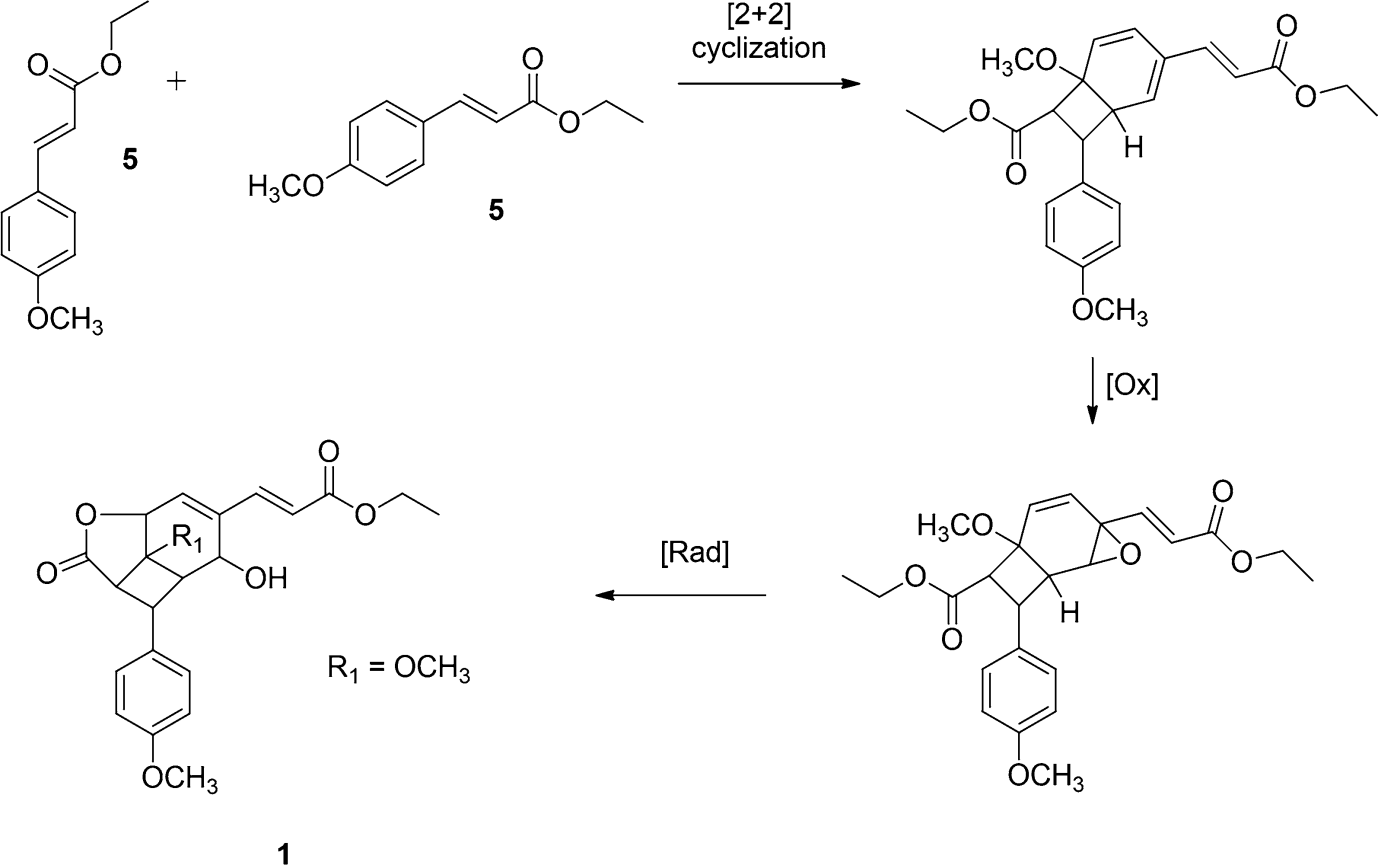
Proposed biosynthetic pathway of **1**

The photochemical generation of **1a** [16] provided a conceptual framework for proposing a plausible biosynthetic pathway for compound **1**, a new natural product derived from *K. galanga*. Given that ethyl 4-methoxycinnamate is a major constituent of *K. galanga*, it is postulated as the precursor (Fig. 2). Exposure to natural sunlight during plant growth or post-harvest drying processes may facilitate [2 + 2] cycloaddition [21] between the trans double bond of compound **5** and the aromatic ring, followed by epoxidation and subsequent ring opening, ultimately forming the lactone through double bond migration and carboxylate conjugation. Based on its structural features and proposed biosynthesis, compound **1** can be classified as a phenylpropanoid dimer rather than a lignan, since its formation involves photochemical dimerization rather than oxidative coupling of monolignols [22].

**Fig. 3:**
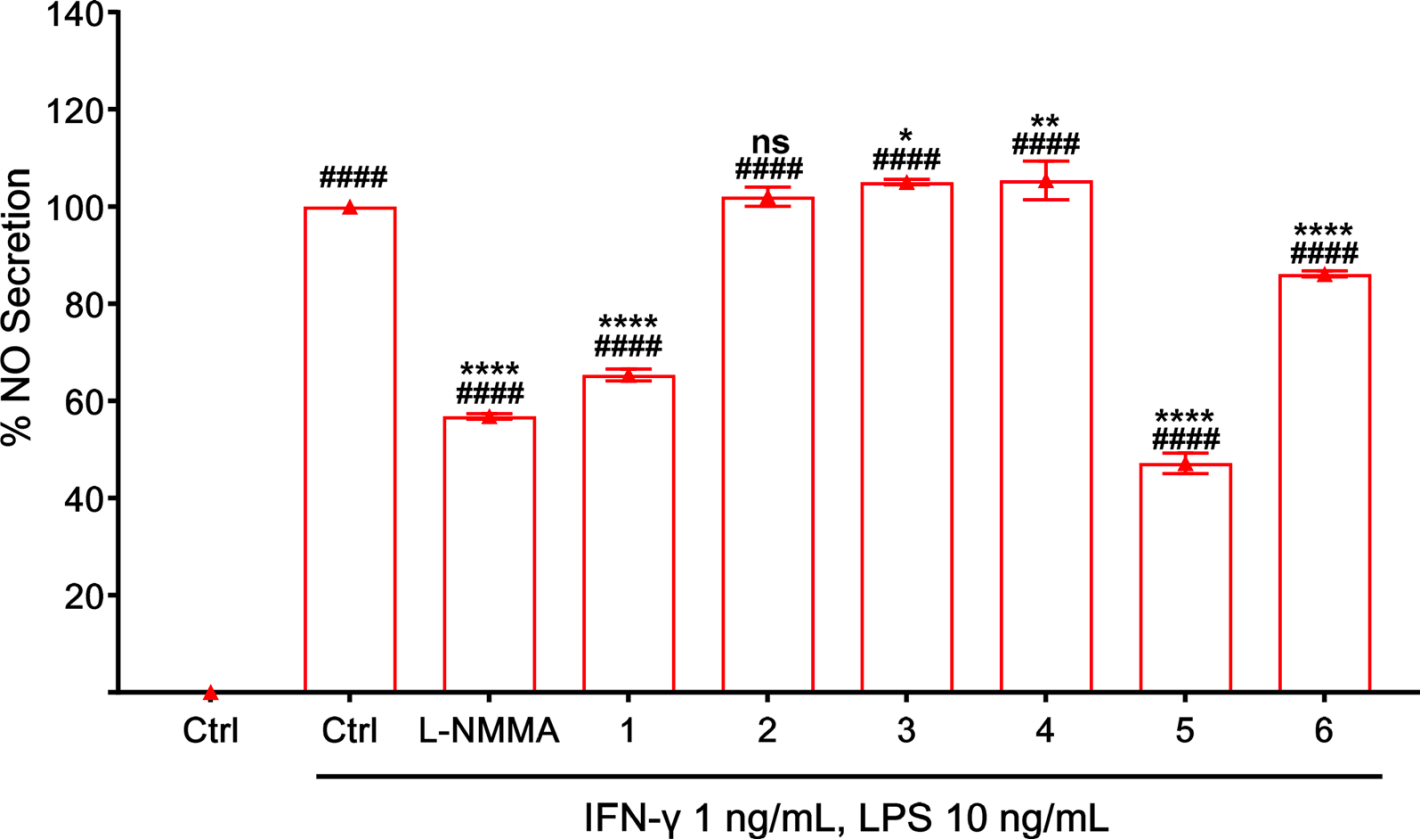
Effect of isolated compounds on NO secretion in RAW264.7 cells. Concentration of sample is 40 μM except for L–NMMA (100 μM). All the results are presented as the mean ± SD (n = 3). Statistical analysis was conducted using one way ANOVA (Tukey’s test). Significance difference (^####^*p* < 0.0001) compared with vehicle control group, whereas (**** *p* < 0.0001, ** *p* < 0.01, * *p* < 0.05) compared with LPS treatment group

Inflammation is a vital physiological defense mechanism involving intricate networks of cellular and molecular mediators; however, its dysregulation is implicated in the onset and progression of numerous chronic diseases, highlighting the need for safe and effective anti-inflammatory therapies [23]. Among these mediators, nitric oxide (NO) plays a dual role—functioning as an anti-inflammatory agent under normal physiological conditions, but contributing to pathological inflammation when produced in excess [24]. *K. galanga* demonstrates potent analgesic and anti-inflammatory effects, mediated by its bioactive constituents that suppress inflammatory signaling pathways [25]. Previous studies demonstrated that ethyl 4-methoxycinnamate (**5**), the principal compound in *K. galanga*, exhibited dual COX-1 and COX-2 inhibitory activity at 1.12 µM and 0.83 µM, respectively [19]. Moreover, another reported its inhibitory activity on IL-1 and TNF-α with IC_50_ values of 166.4 µg/mL and 96.84 µg/mL, respectively [26]. In alignment with these reports, compound **5** in this study inhibited NO production with an IC_50_ of 12.2 ± 4.0 µM (Table 3). Compounds **3**–**4** and **5** showed notably different anti-inflammatory activities, with compound **5** being the most potent. This enhanced activity is likely due to its conjugated double bond, which may improve interaction with inflammatory targets such as iNOS. Structural features like unsaturation appear to significantly influence anti-inflammatory potency, as supported by this result. Intriguingly, the newly identified phenylpropanoid bearing cyclobutane ring, compound **1**, also exhibited significant NO inhibitory activity with an IC_50_ of 23.1 ± 6.4 µM, suggesting promising anti-inflammatory potential (Fig. 3).

**Table 3:** The IC_50_ values of isolated compounds

Compounds	IC_50_ (μM)
**1**	23.1 ± 6.4
**2**	ND
**3**	ND
**4**	ND
**5**	12.2 ± 4.0
**6**	43.8 ± 2.1

Mean ± SD, n = 3

## Conclusion

This study reports on the isolation and structural elucidation of kaemphenolide (compound **1**), a cyclobutane-containing phenylpropanoid from *K. galanga*. The compound expands the chemical diversity of this medicinal plant and showed promising anti-inflammatory potential by inhibition of NO production (IC_50_ 23.1 µM), supporting its traditional anti-inflammatory use. Notably, the rigid and conformationally constrained nature of the cyclobutane ring may contribute to its enhanced bioactivity. Its proposed biosynthesis via [2 + 2] cycloaddition highlights the role of photochemical processes in natural product formation. These findings indicate that *K. galanga* represents a promising natural source for the development of therapeutic agents targeting inflammatory disorders.

## Experimental section

### General

NMR spectra were obtained on a Bruker Ascend 600 (600 MHz), HPLC was performed on a Shimadzu LC-10Advp series (pump; LC-10AD VP, Diode Array Detector (DAD); SPD-M-10A VP, Column oven; CTO-10A) and JASCO-2000 Plus series (pump; PU-2080 Plus, DAD; MD-2018 Plus, Column Oven; CO-2060 Plus). The Mass Spectra were measured by LC/MS Thermo Scientific; LC Dionex Ultimate 3000; LTQ Orbitrap Elite. Recycling preparative HPLC was performed on a Japan Analytical Industry LC-908W. The IR data was collected by FT/IR-460 plus JASCO. Organic solvents (methanol, *n*-hexane, ethyl acetate, chloroform, *n*-butanol, and dehydrated-pyridine) were purchased from Fujifilm Wako Pure Chemical Corporation. LC/MS grade solvent (0.1% Formic acid in acetonitrile and 0.1% Formic acid in water) were purchased from Thermo Fisher Scientific. Silica gel 60 (Spherical) 100-20 μm was purchased from Kanto Chemical Co., Inc. Sephadex LH-20 was obtained from Sigma-Aldrich Co. LLC., 4-nitrophenyl chloroformate from Tokyo Chemical Industry Co. LTD.

The dried *K. galanga* was purchased from Nihon Funmatsu Yakuhin Co., Ltd.

### Extraction and isolation

The extraction protocol commenced with 1.48 kg of dried *K. galanga* rhizome powder subjected to exhaustive methanol extraction under reflux conditions (2 hours × 2 repetitions). The combined filtrates were concentrated under reduced pressure to yield 158.89 g of crude methanol extract. Subsequent fractionation involved suspending the crude extract in water followed by sequential partitioning with *n*-hexane and ethyl acetate, yielding two distinct fractions: an *n*-hexane-soluble fraction (KG-H, 94.2 g) and an ethyl acetate-soluble fraction (KG-E, 6.41 g).

The KG-H fraction underwent methanol crystallization, producing compound **5** (19.06 g) as primary crystals. The remaining filtrate (10 g) was fractionated through primary silica gel column chromatography (7 cm i.d.) using a gradient of *n*-hexane–ethyl acetate (started with ratio of 10% ethyl acetate continued to 100%), yielding nine subfractions (FR-1–1 to FR-1–9). Further purification of these subfractions via secondary silica gel chromatography (5 cm i.d.) with hexane–ethyl acetate gradient (started with ratio 30% of ethyl acetate continued to 60%, 80%, 100% and 30% for over 35 min) yielded FR-2–3 and FR-2–7. Final purification of FR-2–7 by reversed-phase HPLC (Waters XBridge Prep C18 column, 5 μm, 10 × 250 mm; acetonitrile–water gradient at 2.5 mL/min, 40 °C) afforded compound 2 (0.6 mg).

The KG-E fraction was subjected to comprehensive silica gel chromatography (8 cm i.d.) using *n*-hexane–ethyl acetate–methanol gradient system (started with ratio of 10% to 100% of ethyl acetate and continued to 1%-10% of methanol), generating 32 fractions. Pooled fractions FR-22 to FR-25 were further resolved through silica gel chromatography (3 cm i.d.) with chloroform–methanol gradient (started from 100 to 50% of chloroform), followed by reversed-phase HPLC purification (identical column parameters with methanol–water mobile phase) to yield FR-3–2, compound **1** (4 mg), and compound **6** (2.5 mg). Final purification of FR-3–2 by normal-phase HPLC (Kanto Chemical) Mightysil Si60 column, 5 μm, 10 × 250 mm; *n*-hexane–ethyl acetate gradient (started with ratio 30% of ethyl acetate continued to 60%, 80%, 100% and 30% for over 35 min) at 2.5 mL/min, 40 °C) afforded compounds **3** (47.4 mg) and **4** (4.9 mg).

### Cell assay

RAW264.7 cells were seeded in 96-well plates at a density of 2 × 10^5^ cells/200 µL per well and incubated for 2 hours at 37 °C in a humidified atmosphere containing 5% CO_2_ using SCA-165DRS (Astec Co. Ltd) CO2 Incubator. Subsequently, cells were stimulated with interferon-γ (IFN-γ, 1 ng/mL) and lipopolysaccharide (LPS, 10 ng/mL) in the presence of test samples dissolved in DMSO at various concentrations. The plates were further incubated for 16 hours under the same conditions. L-*N*^*G*^-monomethyl arginine (L-NMMA), a known inhibitor of inducible nitric oxide synthase (iNOS), served as the positive control. Following incubation, 100 µL of the culture supernatant was transferred from each well, to which 50 µL of 1% sulfanilamide in 5% phosphoric acid and 0.1% N-1-naphthyl ethylenediamine dihydrochloride were sequentially added [27]. The mixtures were incubated for 15 minutes at room temperature under Light-protected conditions. Absorbance was recorded at 550 nm with a reference wavelength of 655 nm using a xMark microplate reader (Bio-Rad Laboratories, Inc.), and % NO secretion was calculated as described by Miyazawa et al. [28]. Samples exhibiting inhibitory effects on NO production were further evaluated at various concentrations to determine their IC_50_ values.

### Statistical analysis

Statistical analyses were performed using GraphPad Prism version 10. Methods and representative symbols are described in the legends of the figures. Symbols mean significant differences from mean values of indicated numbers of independent experiments. A one-way ANOVA followed by Tukey’s test was used to compare multiple different variables between two experimental groups, respectively. For all analyses, *p*-values below 0.05 were considered statistically significant and were indicated in the legends of the figures.

## Supplementary Information

Supplementary material 1

## Data Availability

Not applicable
