# Supplementary material for: Kaemphenolide: a cyclobutane-bearing phenylpropanoid from Kaempferia galanga L. with nitric oxide inhibitory activity
Source: Nat Prod Bioprospect. 2025 Oct 2;15(1):68. doi: 10.1007/s13659-025-00547-2 (PMC12488551; doi:10.1007/s13659-025-00547-2)
Supplement: Supplementary file 1 — Supplementary material 1 [file 13659_2025_547_MOESM1_ESM.docx]

Supplementary Information

**Kaemphenolide: A Cyclobutane-Bearing Phenylpropanoid from *Kaempferia galanga* L. with Nitric Oxide Inhibitory Activity**

**Syarifatul Mufidah^1,2^, Yusaku Miyamae^3*^, Hiroyuki Fuchino^4^**†**, Nobuo Kawahara^4^**‡

^1^Master’s/Doctoral Program in Life Science Innovation, University of Tsukuba, Japan. ^2^Faculty of Pharmacy, Universitas Ahmad Dahlan, Indonesia. ^3^Institute of Life and Environmental Sciences, University of Tsukuba, Japan. ^4^Research Center for Medicinal Plant Resources, National Institutes of Biomedical Innovation, Health and Nutrition, Japan.

^*^To whom correspondence: [miyamae.yusaku.fw@u.tsukuba.ac.jp](mailto:miyamae.yusaku.fw@u.tsukuba.ac.jp); Phone: +81-29-853-6417

†Present address: Faculty of Pharmacy, Niigata University of Pharmacy and Medical and Life Sciences, Japan.

‡Present address: The Kochi Prefectural Makino Botanical Garden, Japan.


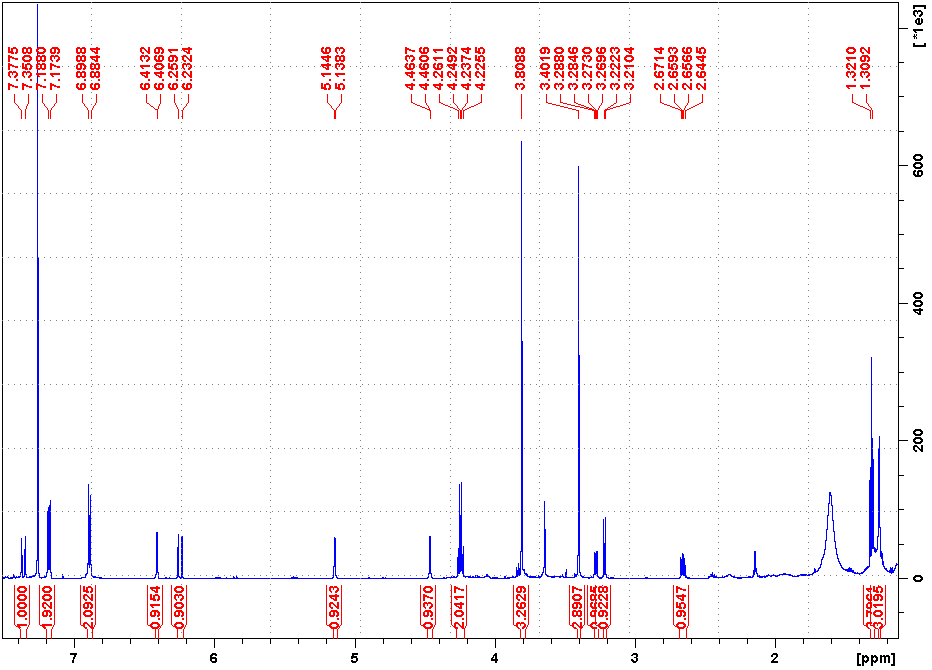


**Figure S1.** ^1^H NMR spectrum of **1**

**
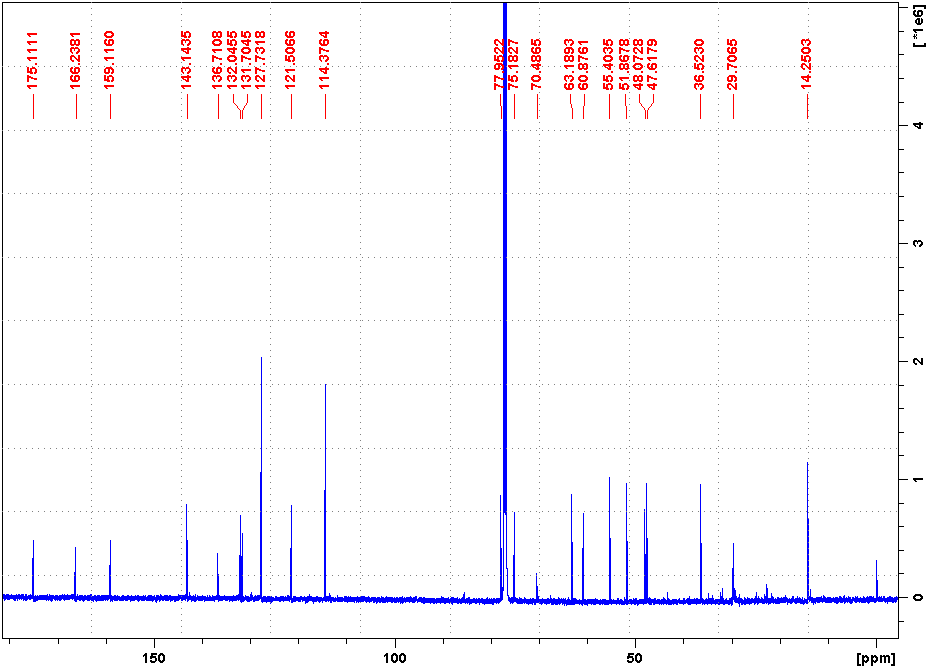
**

**Figure S2.** ^13^C NMR spectrum of **1**


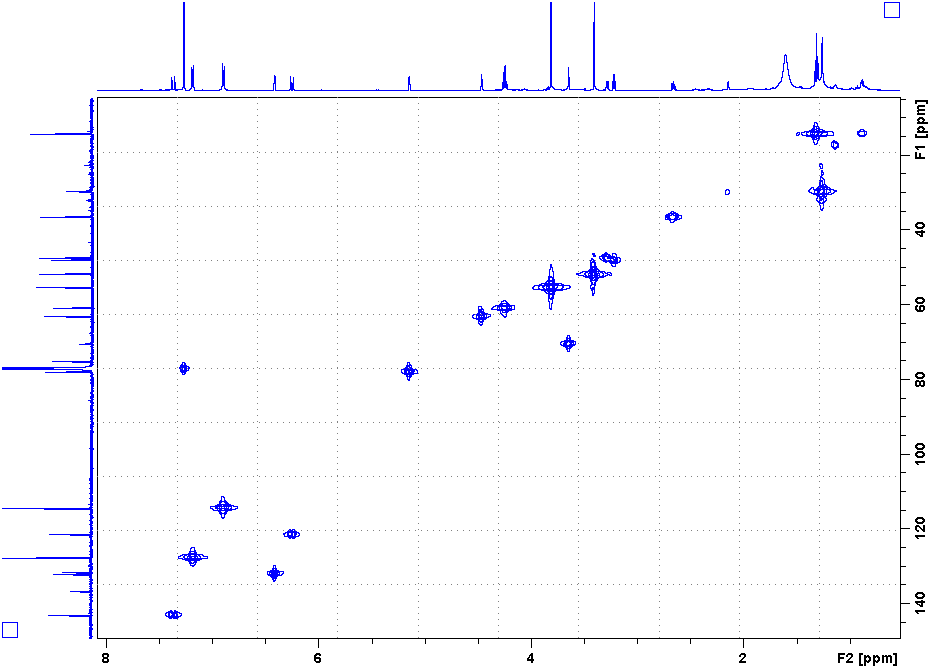


**Figure S3.** HMQC correlation of **1**

**
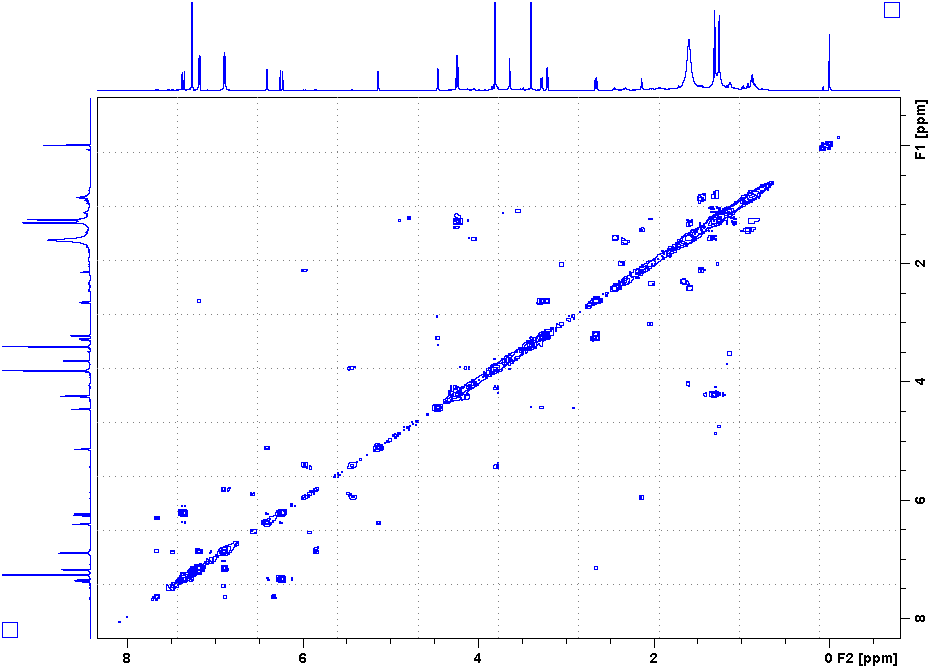
**

**Figure S4.** ^1^H-^1^H COSY correlation of **1**

**
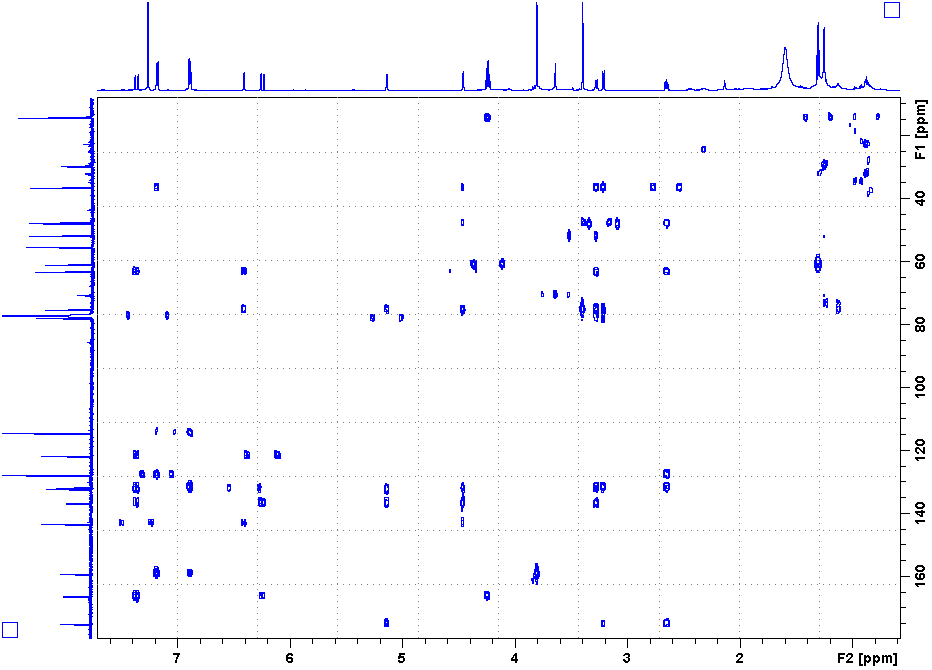
**

**Figure S5.** HMBC correlation of **1**

**
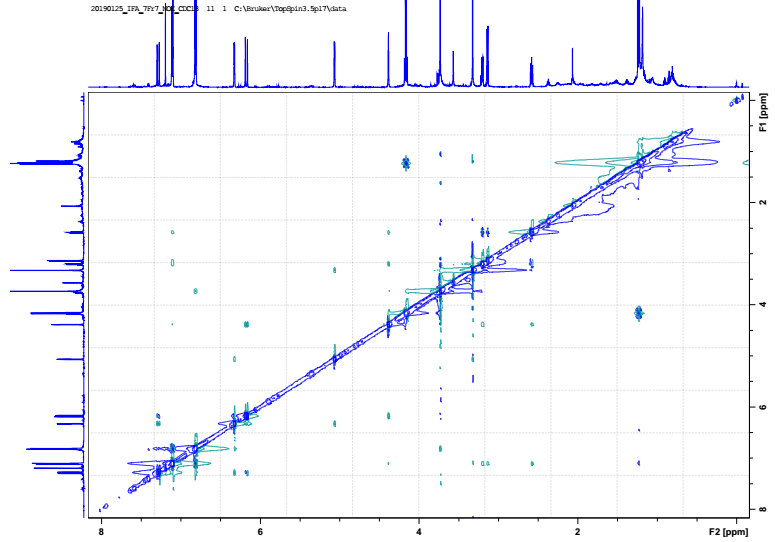
**

**Figure S6.** ^1^H-^1^H NOESY correlation of **1**


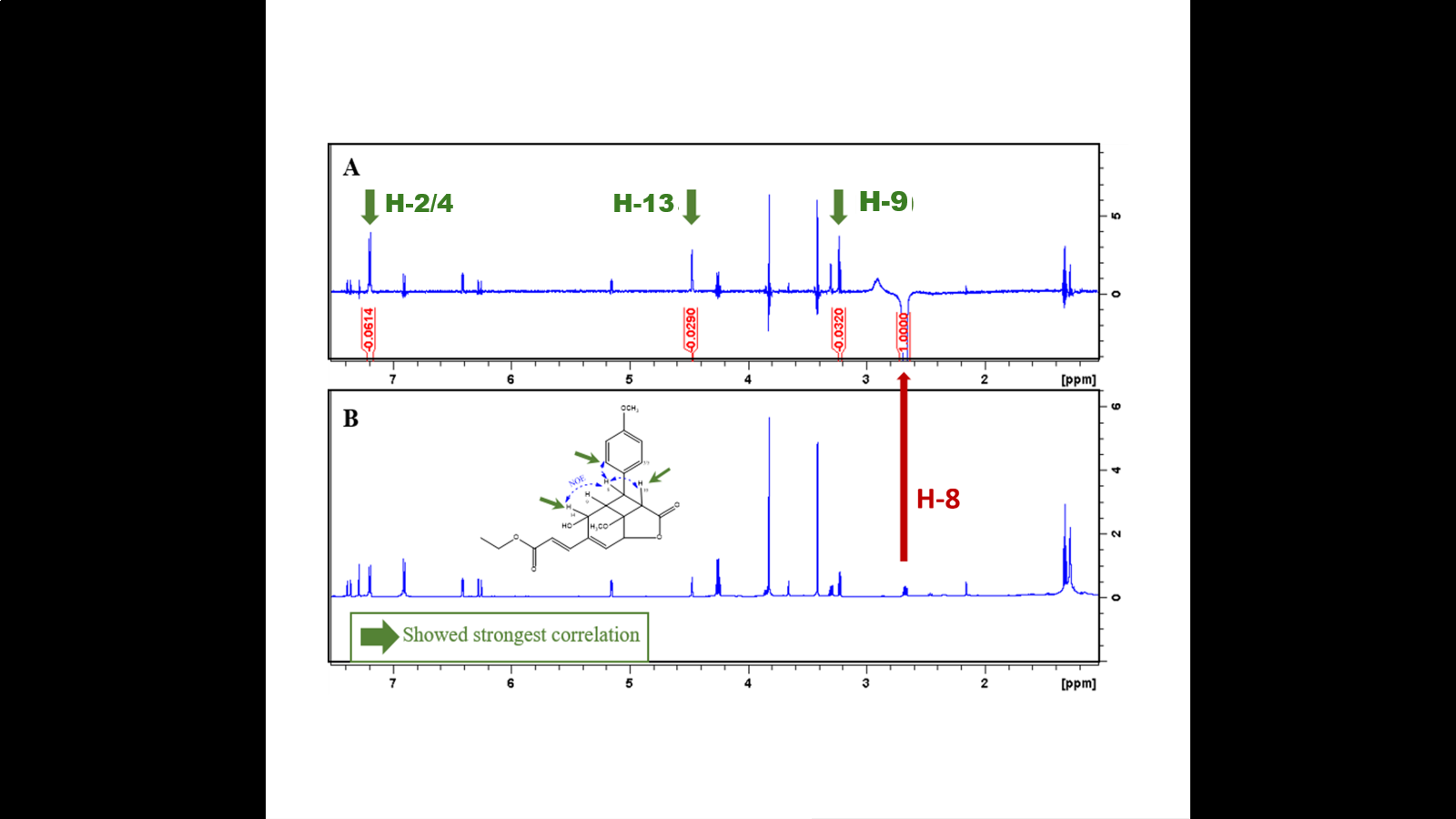


**Figure S7.** (A) Selective 1D-difference between NOE of H-8 and (B) ^1^H NMR spectrum of **1**.





Figure S8. Effect of isolated compounds on NO secretion in RAW264.7 cells with different concentration. Concentration of L-NMMA is 100 μM. All the results are presented as the mean + SD (n = 3). Statistical analysis was conducted using one way ANOVA (Tukey’s test). Significance difference (^####^*p* < 0.0001) compared with vehicle control group, whereas (**** *p* < 0.0001, ** *p* < 0.01, * *p* < 0.05) compared with LPS treatment group.
